# The improving strategies and applications of nanotechnology-based drugs in hepatocellular carcinoma treatment

**DOI:** 10.3389/fbioe.2023.1272850

**Published:** 2023-09-21

**Authors:** Xiangyang Ren, Danyang Su, Doudou Shi, Xiaohong Xiang

**Affiliations:** ^1^ The First Affiliated Hospital of Zhengzhou University, Zhengzhou, China; ^2^ The Ninth Hospital of Xi’an, Xi’an, Shaanxi, China

**Keywords:** nanotechnology-based drugs, hepatocellular carcinoma, treatment, applications, improving strategies

## Abstract

Hepatocellular carcinoma (HCC) is one of the leading causes of tumor-related death worldwide. Conventional treatments for HCC include drugs, radiation, and surgery. Despite the unremitting efforts of researchers, the curative effect of HCC has been greatly improved, but because HCC is often found in the middle and late stages, the curative effect is still not satisfactory, and the 5-year survival rate is still low. Nanomedicine is a potential subject, which has been applied to the treatment of HCC and has achieved promising results. Here, we summarized the factors affecting the efficacy of drugs in HCC treatment and the strategies for improving the efficacy of nanotechnology-based drugs in HCC, reviewed the recent applications’ progress on nanotechnology-based drugs in HCC treatment, and discussed the future perspectives and challenges of nanotechnology-based drugs in HCC treatment.

## 1 Introduction

China has the largest population of hepatocellular carcinoma (HCC) in the world and most HCC patients are already in the advanced stage when diagnosed (Vogel et al., 2022). The prognosis of early HCC is better through surgical resection and the treatment of advanced HCC is mainly local treatment and drug therapy. Currently, the drugs used in the treatment of HCC mostly include chemotherapy, immunotherapy, targeted therapy and combination therapy. Due to the use of these effective drugs, the death rate of HCC is decreasing by 2% per year (Toh et al., 2023). With the deepening of research, it has been found that the efficacy of drug therapy for HCC is affected by many factors, such as primary and secondary drug resistance, tumor microenvironment (TME), etc (Bao and Wong, 2021). Therefore, improving the therapeutic effect of advanced HCC is an important factor in improving the prognosis of HCC.

Currently, the drugs commonly used in the treatment of HCC mainly include proteins/peptides, small molecules, and nucleic acids. The former mainly includes multi-kinase inhibitors such as sorafenib and monoclonal antibodies, while the latter approved for clinical applications includes MTL-CEBPA (Setten et al., 2018). Many other genes, siRNAs, shRNAs, miRNAs, etc. have been identified as candidate therapeutic targets for HCC. Along with the successful application of mRNA vaccines such as COVID-19 vaccines (Hussain et al., 2022), more new drugs such as cancer vaccines have gradually entered the stage of preclinical or clinical trials (Gote et al., 2023). At present, cancer vaccines could be produced based on tumor cells or immune cell, peptides, viral vectors, and nucleic acids (Chehelgerdi and Chehelgerdi, 2023). mRNA vaccine has attracted much attention for its advantages of high expression efficiency and range, no genome insertion, etc. In addition, mRNA transcription *in vitro* (IVT) could be used for vaccine mass production due to its simple and fast reaction (Miao et al., 2021). Cancer mRNA vaccines targeting tumor antigens show great potential in the personalized and precise treatment of cancer (Precision medicine meets cancer vaccines, 2023). For example, PROVENGE, an immune cell-based vaccine, has been approved by the US FDA to treat hormone-refractory prostate cancer (Cheever and Higano, 2011). Studies have shown that storage conditions, formulation, stability *in vivo*, targeting, blood concentration, target concentration and other factors are important factors affecting the therapeutic effect of drugs (Vitharana et al., 2023; Woodring et al., 2023). In the process of new drug research exploration, researchers have conducted more investigations for these factors and made great progress. For example, mRNA generated by the IVT method often contains dsRNA impurities, which could inhibit translation and induce mRNA degradation, while it could activate IFN-Ⅰ signaling mediated immune responses (Chehelgerdi and Chehelgerdi, 2023). And the purified IVT mRNA by high-performance liquid chromatography (HPLC) and VSW-3 RNAP could effectively solve this problem (Xia et al., 2022; Chehelgerdi and Chehelgerdi, 2023). To increase the stability of nucleic acid drugs, nucleic acid drugs are encapsulated to avoid contact with a complex internal environment (Sun et al., 2023). mRNA was modified and codon-optimized to improve mRNA drug expression in target cells (Chehelgerdi and Chehelgerdi, 2023). Currently, the FDA has approved a vaccine against the hepatitis B virus, which can cause HCC (Guo et al., 2013) and more cancer mRNA vaccines are expected to be approved for clinical cancer treatment, including HCC in the future. However, how to maximize the therapeutic effect of drugs and reduce toxic side effects still needs to be optimized and explored. If these problems are solved, the prognosis of HCC patients is expected to be improved significantly.

**FIGURE 1:**
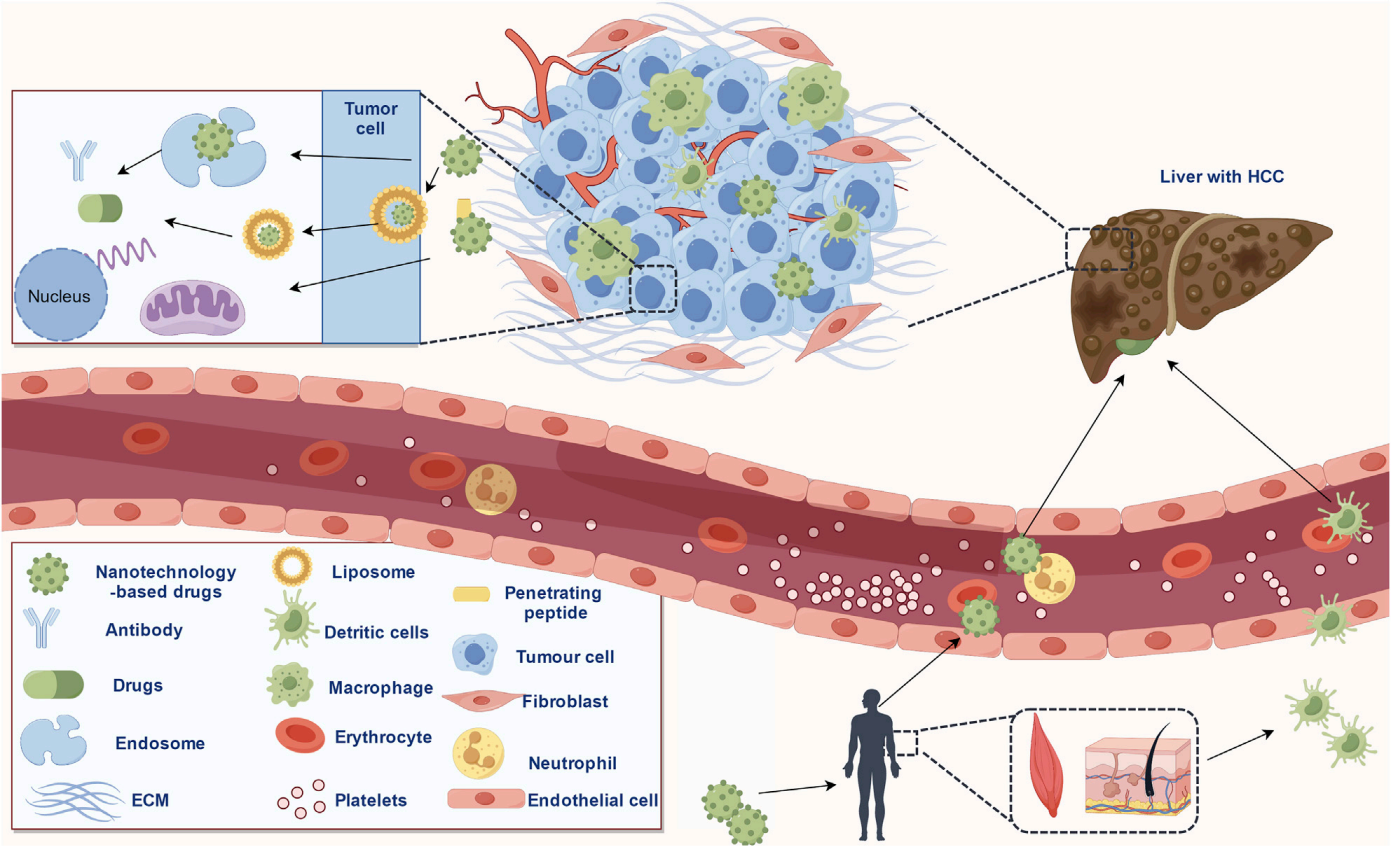
Schematic representation of the nanotechnology-based drugs delivery in HCC treatment (copyrights obtained from Figdraw).

Nanomedicine is the application of the principles and methods of nanoscience and technology to medicine. Nanomaterials show great potential in the field of cancer therapy due to their properties of nanoscale size, good biocompatibility, response to a specific stimulus, large surface area, and multiple functional modifications (Sohrabi Kashani and Packirisamy, 2021). A schematic representation of nanotechnology-based drug delivery in HCC treatment is shown in Figure 1. Studies have shown that most oral or intravenous drugs were nonspecifically ingested and cleaned by the liver, which is associated with their rich reticuloendothelial phagocytosis system (RES) (Roy et al., 2022). Thus, nanotechnology-based strategies of promoting the binding of drugs to target-specific interaction of serum proteins (attention should be paid to avoiding excessive aggregation of molecules in the blood) and facilitating the separation of drugs from plasma proteins at the HCC site can effectively reduce the non-specific clearance of drugs (Kim et al., 2023). In addition, the size, surface charge, and other characteristics of nano-delivery vectors could affect liver uptake. For example, nanoparticles with a diameter of 60 nm tend to be enriched rather than eliminated in the liver (Xu et al., 2022). Furthermore, the real-time imaging function of nanomaterials can detect the distribution of drugs in tumors, and the response characteristics such as pH and glutathione (GSH) of nano-delivery vectors can significantly reduce the side effects and drug resistance (Yang et al., 2021). In recent years, nanotechnology-based drugs have made many important advances in the treatment of HCC and show great potential. Here, we reviewed the factors affecting the efficacy of drugs in HCC, and the contribution of nanotechnologies in drug properties such as stability and targeting in HCC. The applications of nanotechnology-based drugs in the treatment of HCC were briefly reviewed. Finally, we pointed out the limitations of the current study.

## 2 Factors affecting the efficacy of drugs in HCC

### 2.1 Drug stability

For protein/peptide drugs, the method of administration affects the stability of the drug. For example, oral use results in low drug absorption rates and insufficient doses to treat HCC. And increasing the dose could lead to increased side effects such as kidney dysfunction (Haddadzadegan et al., 2022). While intravenous administration may cause allergic reactions (Lee, 2022). In addition, maintaining protein structure is particularly important for drugs that rely on antigen-antibody or receptor-ligand binding (Lee, 2022). The stability of protein structure is affected by many factors, such as deamidation (Gupta et al., 2022). Deamidation is a common post-translational modification of proteins and also occurs naturally during the preservation of proteins or peptides. The deamidation reaction could convert the amino residues of aspartic acid (Asn) to carboxylic acid, which in turn converts aspartic acid to aspartic acid or beta-aspartic acid. The deamidation of protein is one of the main reasons leading to the loss of protein activity. It could change the local hydrophilicity of proteins, affect the charge distribution of proteins, change the spatial structure of proteins, and thus reduce the activity of proteins. For recombinant proteins, deamidation has a great impact. On the one hand, proteins usually contain a high content of asparagine (∼4%); On the other hand, once the recombinant protein is separated from the host cell and loses its repair mechanism, it may undergo deamidation during long-term storage (Wakankar and Borchardt, 2006). Therefore, it is of great significance to study the deamidation reaction of recombinant protein for its stability and screening of storage conditions. Further, protein oxidation is another important factor in protein inactivation or aggregation (Gupta et al., 2022). Among them, cysteine, methionine, tryptophan, and other amino acid residues are especially easy to be oxidized. Proteolysis is also an important problem in the process of protein purification and preservation. A common view is that proteolytic enzymes cannot be completely removed by the protein purification process, and therefore proteolytic hydrolysis is inevitable during long-term storage in the absence of protease inhibitors (Edwards et al., 2019). Protein concentration is another important factor affecting protein stability. Local microenvironment pH, temperature, salt ion concentration, and so on could also affect the stability of protein/peptide drugs (Edwards et al., 2019).

Nucleic acid drugs include DNA and RNA, with various types of the latter, such as common mRNA, siRNA, and so on (Kulkarni et al., 2021). Gene therapy aims to correct or compensate for gene defects by introducing foreign normal genes into target cells (Sun et al., 2019). Gene therapy drugs include drugs or vaccines based on therapeutic vectors, oncolytic viruses, and cells, such as Gendicine^®^, an adenovirUs-Based drug for non-small cell lung cancer, Reolysin^®^, a reovirus based drug for ovarian cancer, and an oncolytic HSVT-VEC for melanoma (Lundstrom, 2023). Generally, temperature and physical and chemical properties of the solution such as pH are important factors affecting the stability of nucleic acid molecules (Pogocki and Schoneich, 2000). In general, unmodified nucleic acid drugs are less efficient at being absorbed by cells and are easily degraded by nucleases that are ubiquitous in cells. RNA molecules do not have the same double helix structure as DNA molecules, making them more susceptible to chemical reactions and physical damage. In addition, the bases in RNA molecules are also more likely to react than those in DNA, further exacerbating the instability of RNA. This also explains why many RNAs that have been identified as candidate therapeutic targets of HCC have not yet been transformed into clinical targets. MRNA, a large class of RNA molecules, could influence not only the transcription of genes at the transcription level but also the proteins that genes express. Factors affecting mRNA stability have been described in the reference (Sun et al., 2023). A series of achievements have been made in the development of mRNA drugs and vaccines, such as the COVID-19 mRNA vaccines. The development of other RNA drugs is still being explored. The introduction of internucleotide bonded phosphate modified oligonucleotides can improve the stability of siRNA and improve the silencing effect of target genes without potential side effects of treatment (Epanchintseva et al., 2022).

### 2.2 Targeting

Targeting of a drug refers to the characteristic that a drug is selectively concentrated in the target organ, target tissue, or target cell (Fang et al., 2023; Marcello and Chiono, 2023). The target location can be divided into three categories, that is, the first level refers to the specific target tissue or organ, the second level refers to the specific target cell, and the third level refers to the specific targets in the cell. The mode of action can be divided into passive targeting, active targeting, and physical and chemical targeting (Xu et al., 2022). Passive targeting is a distribution feature *in vivo* caused by the natural tendency of macrophages to phagocytose drugs or drug-carrying particles when they enter the body. This type of targeted preparation uses lipids, proteins, and biodegradable polymers as carriers to wrap or embed drugs in a microparticle drug delivery system (Sebak et al., 2021). After intravenous injection, the distribution of passively targeted particles *in vivo* first depends on the particle size (Nakamura et al., 2022). The surface properties of the particles play an important role in the distribution, for example, the size of the side chain changes the optimal particle size for the organ to ingest the nanoparticles (Nakamura et al., 2022). Active targeting preparations include modified drug carriers and precursor drugs. After modification, the drug carrier can either avoid phagocytosis of the mononuclear macrophage system, change the natural distribution of drug-carrying particles in the body, or bind to the receptors or antigens of target cells, so that the drug can be delivered to the target area (Xu et al., 2023). Physical and chemical targeting is the use of physical and chemical methods to make the targeted preparation into a specific location play a role, such as magnetic targeting, embolic targeting, and thermal and pH-sensitive targeting (Yan et al., 2022).

Traditional chemotherapy drug targeting was poor due to their passive targeting, therefore, side effects and toxicity are relatively large (Kundu et al., 2019). For example, cisplatin was most distributed in the liver, kidney, large intestine, and skin after intravenous injection (Harrach and Ciarimboli, 2015). After a single medium or high dose, occasionally mild reversible renal dysfunction and trace hematuria may occur. Irreversible renal dysfunction could occur with repeated use of multiple high doses for a short period (Ghosh, 2019). In severe cases, tubular necrosis could lead to anuria and uremia (Ghosh, 2019). Due to the in-depth understanding of the drug delivery system and tumor microenvironment, researchers are currently working to improve the active targeting and physical and chemical targeting of drugs, to improve the efficacy of drugs and reduce the side effects of drugs and toxicity to non-target sites. For instance, the oral colonic localization drug delivery system could avoid the destruction or release of drugs in the digestive tract and release drugs into the human colon for local or systemic therapeutic effects (Teruel et al., 2020).

### 2.3 Other factors

The properties of nanodrug delivery vectors are important factors affecting drug delivery, such as size, shape, surface charge, and so on (Wang et al., 2022). Nanoparticles in the 100–200 nm diameter range ooze through tumor vascular fenestrations (EPR effect) and escape liver and spleen filtration; As the size increased to more than 150 nm, more and more nanoparticles were encapsulated in the liver and spleen; Small size nanoparticles (<5 nm) are filtered out by the kidney, which leads to drug safety problems and lower concentration of the drug at the target site (Nakamura et al., 2022). For shape, unlike spherical nanoparticles, non-spherical particles, such as those with a disk-like geometry, are more likely to produce tumbling and oscillating effects in the vascular system, greatly increasing the nanoparticle’s tendency to make contact with the cell wall as well as the EPR effect (Blanco et al., 2015). For surface charge, zeta potential is an important index to evaluate the stable dispersion of nanoparticles in the medium. If the Zeta potentiometer detects a negative value, it means that the nanoparticle as a whole shows a negative charge, which is called a negative charge on the surface of the particle, and conversely, it is a positive charge. Positively charged particles are more easily taken up by macrophages in the lungs, liver, and spleen. Neutral and slightly negatively charged nanoparticles have a longer cycle life and less accumulation in the above organs (Blanco et al., 2015). In addition, the surface modification of nanoparticles such as PEG modification affects their surface charge (Samaridou et al., 2020). After contact with serum, a large amount of protein can be adsorbed on the surface of nanoparticles to form a protein crown. The presence of protein crowns could reverse the charge state of positively charged nanoparticles and affect the dispersion of nanoparticles in the biological environment (Docter et al., 2015). The protein crown has a dual function, not only blocking the binding of nanoparticles with non-specific components but also helping to improve the targeting of nanoparticles. For example, Hb-DOXM@Cel, carrying doxorubicin-carrying micelles, hemoglobin crown, and celecoxib, combined with endogenous plasma contact to achieve the targeting of M2 tumor-associated macrophages (TAM), which not only alleviates the hypoxia of tumor microenvironment and kills tumor cells, but also collaborates in reprogramming M2 TAM. The tumor microenvironment was reconstructed into an immune-stimulating microenvironment to enhance the antitumor effect of cytotoxic T lymphocytes (Sun et al., 2022).

**FIGURE 2:**
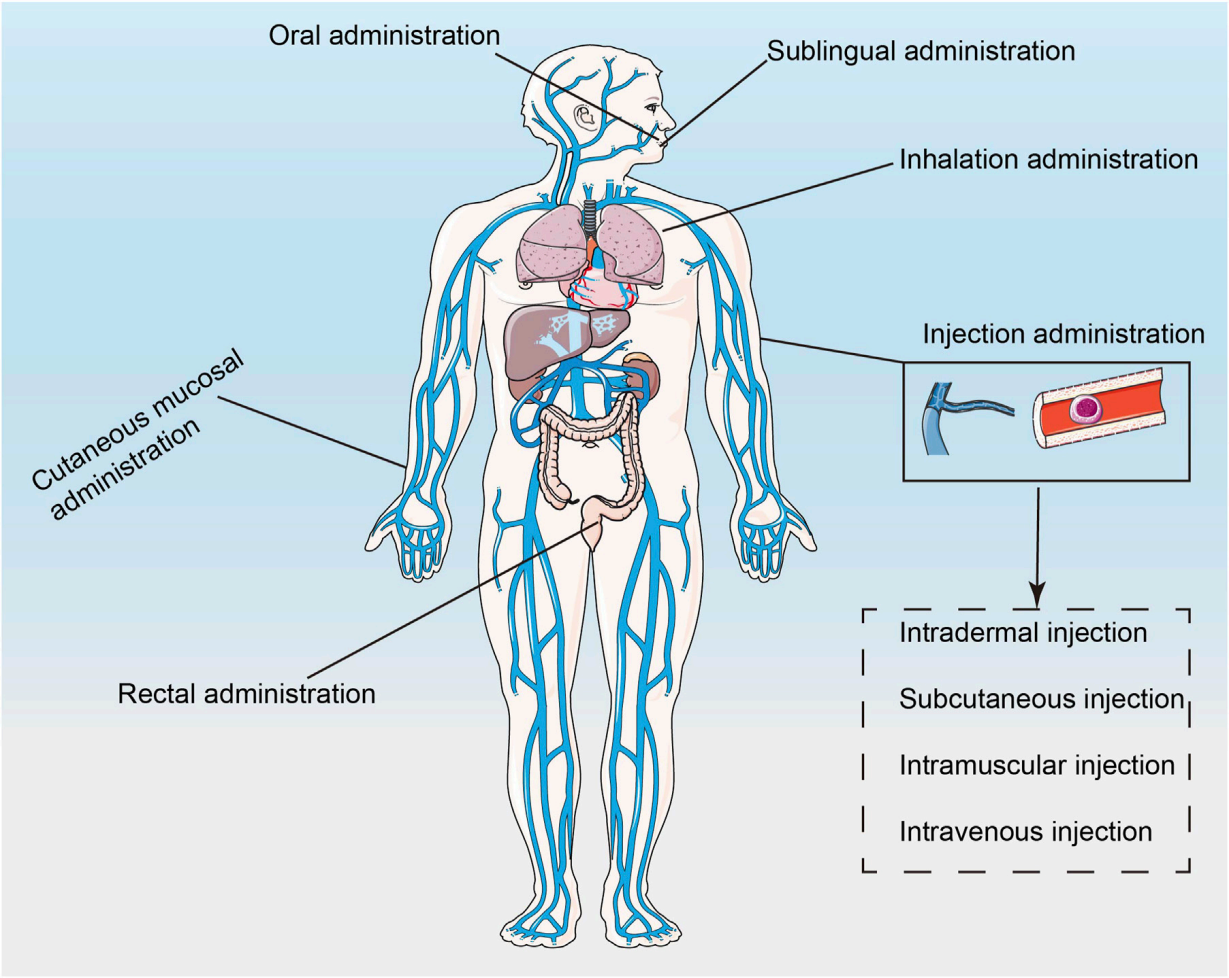
The schematic of how nanotechnology-based drugs enter the body (Xiang et al., 2022).

Nucleic acid drugs, especially RNAi drugs, need to cross the cell membrane to reach the target site and act on the mRNA in the cytoplasm or nucleus, which is difficult to deliver. Although chemical modifications could solve the problems of stability and immunogenicity, small nucleic acid drugs still cannot play a role if they cannot enter the cell to achieve endocytosis (Xin et al., 2017; Kiaie et al., 2022). Traditionally, nucleic acid drugs are transfected using non-viral or viral vectors, and the latter include adenoviruses, adeno-associated viruses, lentiviruses, or retroviruses (Mohammadinejad et al., 2020; Hosseinkhani et al., 2023). Liposomes and electrotransformation are effective *in vitro* rather than *in vivo*. Viral vectors are less efficient, more immunogenic, lack cell type specificity, and carry the risk of genetic mutation, which could lead to toxicity and side effects (Hosseinkhani et al., 2023). In addition, after entering the body, drugs are easily regulated with plasma proteins and then removed from the body. The physicochemical properties of blood affect the half-life and blood concentration of drugs (Zaman et al., 2019; Valencia-Lazcano et al., 2023). The concentration and maintenance time of the drug at the target site are also important factors affecting the efficacy of the drug (Bayer, 2023). Since tumors are heterogeneous diseases, the TME could significantly affect drug efficacy (He et al., 2023). In addition, the mode of administration could also affect the efficacy of the drug (Figure 2). Cancer vaccines are often injected subcutaneously or intramuscularly because these two types of injection could improve the efficiency of antigen presentation (Yi et al., 2023).

In addition, after the drug enters the target organ, how to effectively enter the cell and be transported to the target organelle in the cytoplasm is also an important factor affecting the efficacy of the drug. Effective methods such as electroporation and vector transfection are limited *in vivo*. It is difficult for large molecules such as monoclonal antibodies to enter cells. Drugs can enter cells through endocytosis or direct membrane displacement (Lee et al., 2019). However, the endosomal pathway often leads to the degradation of the delivered substance. Secondly, the release of endosomal contents is also an important factor that directly affects the efficacy of drugs. For example, mRNA drugs can start translation after being released into the cytoplasm by endosomes (Hussain et al., 2022), and gene therapy drugs can play a role after being released into the target chamber by endosomes (Roma-Rodrigues et al., 2020). Proteins and other biomacromolecules cannot escape endosomes by themselves, so other strategies such as nanocarriers with microenvironment responsiveness are needed to promote endosome escape (Lee et al., 2019). Direct membrane displacement utilizes the hydrophilic transformation of lipids and the electrostatic interaction of drugs or carriers with the cell membrane, which leads to transient membrane destruction conducive to drug entry (Lee et al., 2019).

## 3 Current strategies for improving the properties of nanotechnology-based drugs

### 3.1 Current nanotechnology-based strategies for improving drug stability

Given the vulnerability of drug structure, the conventional structural modification strategies of drugs were introduced in the review (Cheng et al., 2023). Here, we mainly summarized the protection strategies of nanotechnology for drug stability, including the encapsulation of drugs by nanomaterials, improving surface characteristics, and modifying nanomaterials. We discuss how these strategies protect drug stability.

**FIGURE 3:**
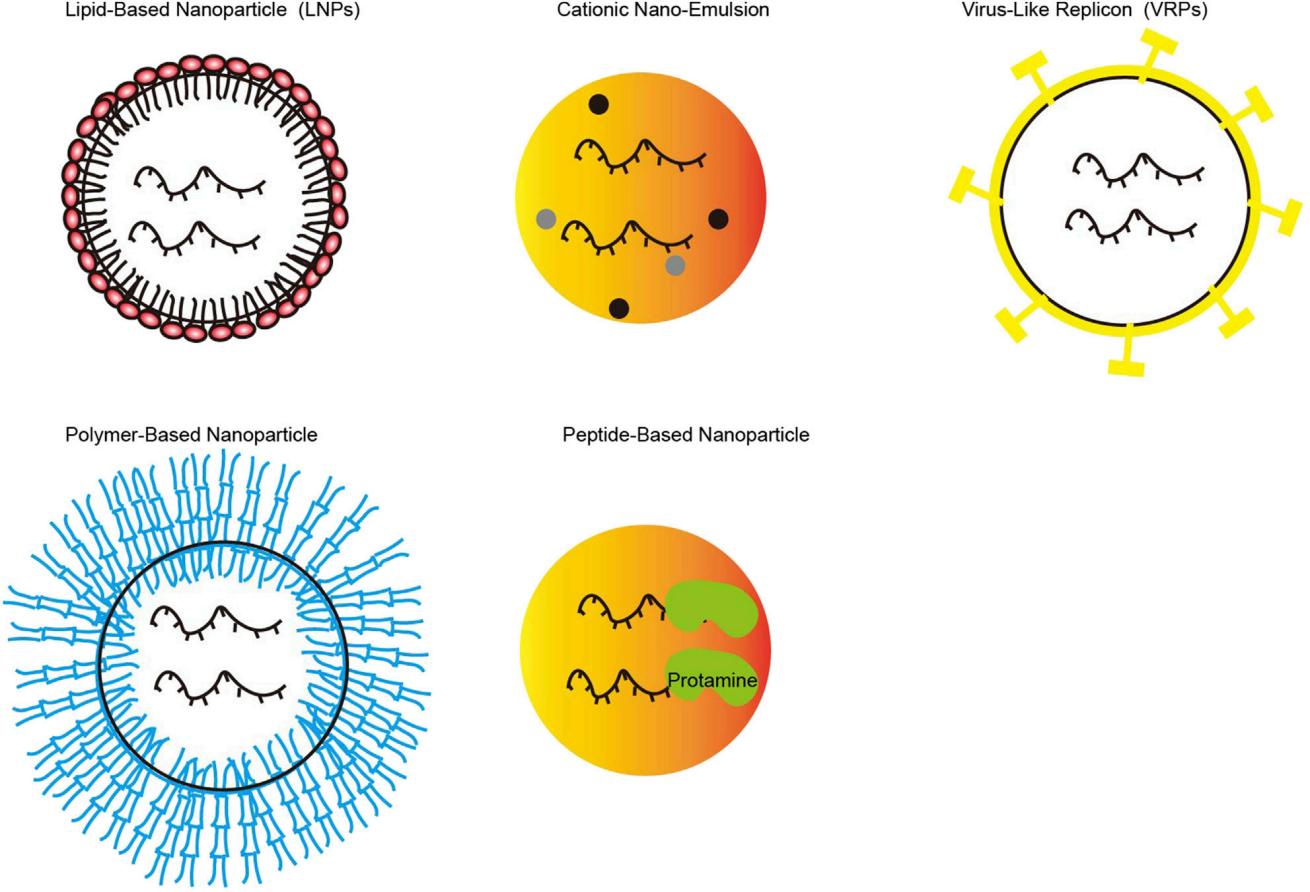
Various of nano-delivery vectors for mRNA vaccines (Picture was adapted from (Hussain et al., 2022)).

Encapsulation of drugs using nanotechnology reduces the chance of deactivation or degradation. For example, the negative charge carried by miRNA is encapsulated in nanoparticles, reducing RES-mediated miRNA clearance (Roy et al., 2022). At present, the commonly used nano-drug delivery carriers are inorganic, organic, biomimetic, and hybrid nanoparticles. The former mainly includes gold nanoparticles, magnetic nanoparticles, quantum dots (QDs), graphene-based, silica-based, and calcium carbonate nanoparticles. And Figure 3 represents the nano-delivery technologies for mRNA vaccines. The latter mainly consists of protein cages such as ferritin and albumin cages (Aljabali et al., 2022), polymer and lipid groups. And polymers could be classified according to whether their components are synthetic or natural (Yan and Zhou, 2023). Lipid groups are divided into nanobodies such as cationic liposomes and liposomes such as exosomes (Tenchov et al., 2021). The properties of these types of nanoparticles have been described in many pieces of literature and have not been described here.

In addition, modifying the surface of nanomaterials and reasonably designing the particle size of nanomaterials are also important strategies to maintain the stability of drugs (Szabo and Zelko, 2015). Polyglycol-lipids modified on the nanoparticles acted as a spatial barrier, preventing the binding of plasma proteins to prolong the drug’s presence in the blood (Samaridou et al., 2020). After the head group of cationic liposomes was replaced with the imidazole group, mRNA stability was increased, mediating endosomal escape and promoting storage time (Dutta, 2021). Increasing the electrostatic action of the drug delivery system is beneficial to increase the stability of the drug. For example, appropriately increasing the negative charge of the lipid nanoparticle drug delivery system could promote mRNA expression (Schoenmaker et al., 2021). When nanomaterials deliver drugs, it is necessary to pay attention to the change of surface charge of nanomaterials after combination. Polyethylenimide (PEI) is a cationic polymer. The composite surface is positively charged after loading mRNA, which makes it easy for the complex to be bound and cleared by negatively charged plasma proteins. Modification with a branched polymer prevents the removal of PEI (He et al., 2020). In addition, surface phospholipatization of amphoteric ions could also reduce the removal of plasma proteins and increase cellular uptake/endosomal escape (Schoenmaker et al., 2021). To mitigate the permanent positive charge of cationic lipids, ionizable lipids have been developed. In addition, biodegradable pH-sensitive ionizable lipids carry no charge in plasma, which reduces plasma protein-mediated clearance (Jorgensen et al., 2023). However, the positive charge in the acidic endosome environment facilitates binding to the endosome membrane and allows the drug to be released (Eygeris et al., 2022). Adjusting the ratio of PEG and ionizable lipids also facilitates the removal of evading drugs from the blood circulation and the release of drugs from the endosome (Schoenmaker et al., 2021). Changing the proportion of side chains such as PEGs changes the optimal particle size for liver uptake (Schoenmaker et al., 2021). Therefore, it is necessary to consider the properties of the nanoparticles and their interaction with the membrane when making formulations and experiments.

Another common approach to maintaining drug stability is to form three-dimensional (3D) drug nanoassemblies, which promote the formation of 3D intramolecular and/or intermolecular structures to block intramolecular cleavage sites (Li G. et al., 2021). For example, high GC content could promote the formation of hydrogen bonds in mRNA chains, which is conducive to the formation and maintenance of mRNA secondary structure (Xian et al., 2023). 3D hydrogels prevent RNase from entering the corresponding cleavage site on mRNA and prolong the half-life of RNA-G4 (RG4) in serum (Ahn et al., 2022).

### 3.2 Current nanotechnology-based strategies for improving drug targeting

Coupling technology is widely used in nanomedicine. This conjugated approach enhances the targeting specificity of drugs by directly covalently binding different components, including lipids, peptides, aptamers, antibodies, and sugars (Li et al., 2017). One of the first attempts was lipid coupling, and N-acetylgalactosamine (GalNAc) coupling is mainly used at present. Compared to lipid molecules, the molecular weight (Debacker et al., 2020) of the coupled delivery system is relatively small, and the endosome escape efficiency of the drug could be enhanced by designing acid-sensitive linkers. Ligand coupling of liver targeting alloxoprotein receptor (ASGPR) is one of the most commonly used coupling delivery systems, and drugs based on GalNac coupling delivery systems have been approved for marketing (Jeon et al., 2022). It is an endocrine receptor that is specifically highly expressed on the surface of the liver cell membrane and is hardly expressed by other cells, so it is also mainly used for liver administration (Kumar and Turnbull, 2023). Although the scope of use is limited to the liver-like lipid molecules, it has the following advantages: 1) good safety, no need for steroid pretreatment; 2) subcutaneous administration could be performed, and patient compliance is better; 3) high delivery efficiency, only 2–5 mg/kg dose could play a role. Lipid coupling has the problem of endosome escape (Sanz-Martinez et al., 2023). Lipid-coupled RNA could form a polymer similarly to low-density lipoprotein (LDL), which not only extends the cycle time but also binds to LDL receptors or other receptors to enter cells through endocytosis and improve delivery efficiency, which is more commonly coupled to cholesterol (Khan et al., 2023). Because LDL receptors are highly expressed in the liver, lipid-coupled drug systems are also administered primarily to target the liver, but by local injection. Also, it could enter tissues such as the skin, eyes, and brain. However, RNA drugs that initially use a coupled delivery system often do not function after entering the cell, mainly because the RNA molecules get “stuck” in the internal body, and only about 0.01% of the molecules can escape into the cytoplasm to function. Therefore, endosome escape becomes the rate-limiting step of coupled drug delivery (Feingold, 2000).

The principle of active targeting has been described above, and more results have been obtained in animal experimental models, such as scFv-Z@FRT (Li et al., 2018) that target fibroblast activating protein (FAP) on liver fibroblasts and ADOPSor-NPs that antagonize CXCR4 to avoid HCC cell proliferation and M2 polarization (Chen et al., 2014). However, the *in vivo* environment is relatively complex, such as the mutation or loss of antigens or ligands caused by tumor gene mutation, and the change of antigen conformation on tumor surface influenced by TME, which reduces the targeting of nanotechnology-based drugs. More preclinical data are needed to promote the progress of clinical applications. In addition, some nanoparticles also respond to external stimuli such as ultrasonic stimulation, heat, pH, and light, and these types of nanoparticles could release drugs in a controlled manner, thereby improving the precision and targeting of drug delivery (Liu et al., 2023; Nie et al., 2023), such as pH-sensitive targeting of gastrointestinal and vaginal delivery systems (Abdella et al., 2023), extracellular matrix sensitive GNPs-Dox-Lac (Liu et al., 2018) and magnetic Fe_3_O_4_ nanoparticles (Chen and Hou, 2022) At present, physical and chemical targeted nanodrug delivery systems have shown great potential in animal models, but the physical and chemical properties *in vivo* are dynamic, and how to accurately set the stimulus perception of nanodrug delivery vectors remains to be further studied.

### 3.3 Current strategies to improve nanotechnology-based cytosolic delivery

**FIGURE 4:**
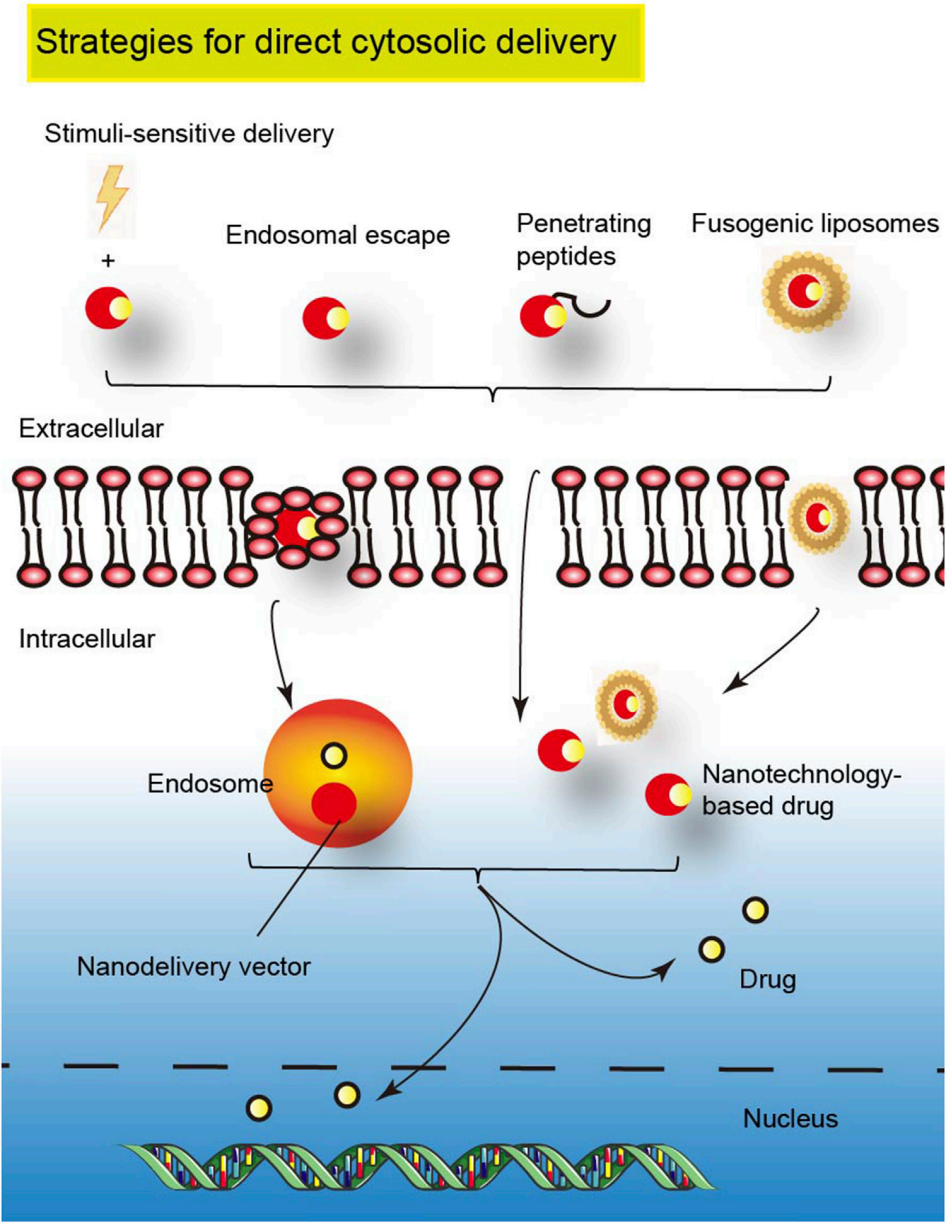
Schematic representation of strategies for cytosolic delivery of nanotechnology-based drugs (copyrights of part elements obtained from Figdraw).

In response to the difficulty of drug entry into cells, the nanocarrier system has been studied as an effective carrier to promote drug uptake by cells. Since the phospholipids of the cell membrane are generally negatively charged, positively charged nanocarriers have been designed and prepared to carry drugs into the endosomes by electrostatic action (Lee et al., 2019). Later, it was found that positively charged nanocarriers easily interact with plasma proteins and are eliminated. Environmentally responsive nanocarriers have also been developed, which carry no charge in the blood and activate the endosomal microenvironment to promote the rupture of endosomal membranes and release drugs. For example, GSH-responsive disulfide-lenalidomide-methoxy polyethylene glycol (LND-DSDA-mPEG) (Liu et al., 2023). However, the acidic environment might have an impact on the release of drugs, such as some protein drugs that function by antigen-antibody or ligand-receptor binding. In addition, how to control the precision of the required stimulus is also a problem that needs to be solved. Except for endosomal escape and stimulus-sensitive delivery, cytosolic delivery strategies of drugs also include penetrating peptides and fusogenic liposomes (Son et al., 2023) (Figure 4), such as penetrating peptides modified on nano-delivery vectors, which can be delivered into cytoplasm in a variety of ways. These cytosolic delivery strategies have been approved or used in the clinic. The future. More research should focus on the combination of multiple cytoplasmic strategies.

## 4 The applications of nanotechnology-based drugs in HCC treatment

**FIGURE 5:**
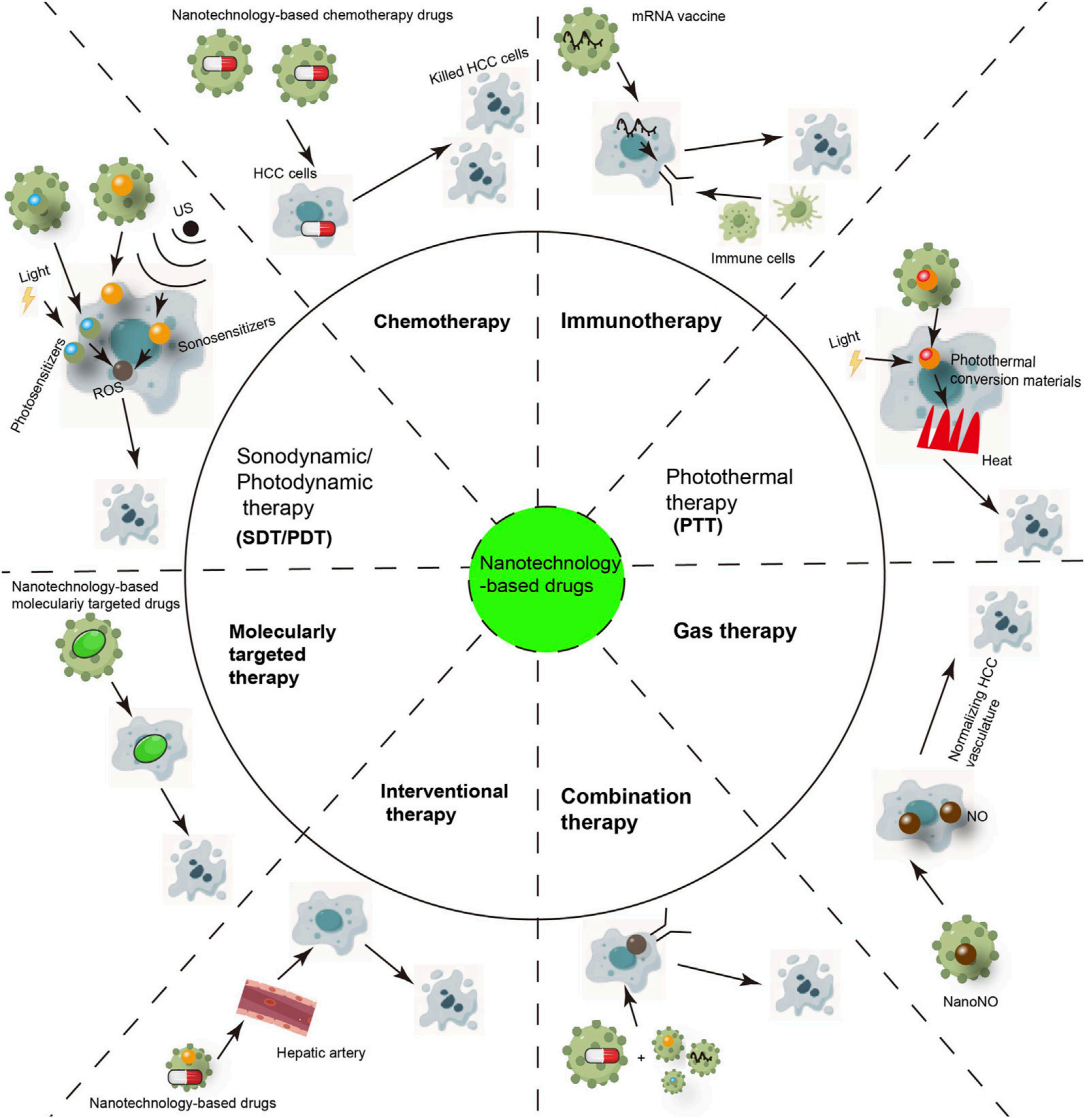
The Applications of Nanotechnology-based drugs in hepatocellular carcinoma treatment (copyrights of part elements obtained from Figdraw).

Nanotechnology-based drugs can be used in various fields of HCC treatment, such as immunotherapy, chemotherapy, transarterial chemoembolization, and targeted therapy (Figure 5). And nanotechnology-based drugs may include existing commonly used targeted drugs, chemotherapy drugs, and immunotherapeutic drugs, as well as new candidate siRNA, mRNA, and miRNA, targeting a certain target or pathway. Next, we discussed the application of nanotechnology-based drugs in the field of HCC treatment.

### 4.1 Chemotherapy

Traditional chemotherapeutic drugs such as doxorubicin, cisplatin, oxaliplatin have unsatisfactory effects on the treatment of HCC, and the toxicity and side effects are obvious (Hou et al., 2022). The toxic side effects of chemotherapeutic drugs are often caused by non-specific ingestion and dose dependence. Therefore, the use of nanotechnology to improve the uptake of target cells and the duration of local blood concentrations of chemotherapy drugs is of great significance for enhancing the efficacy of chemotherapy and reducing the side effects of chemotherapy drugs (Jia et al., 2022). The calcium carbonate nanoparticles were prepared by the microemulsion method. The nanoparticles had 70% drug release at pH 5.5, and the cisplatin and oleanolic acid could promote the apoptosis of HCC cells and reduce the hepatotoxicity caused by cisplatin (Khan et al., 2019). Dual-targeting nanoparticles inhibited cisplatin resistance in HCC, the mechanism was that the metal-organic framework core was modified by nuclear localization sequence, which was conducive to the internalization of tumor cells, and carried cisplatin and NOR1 shRNA; the outer shell was A54 peptide, which could bind to the A54 receptor on the surface of HCC cells. Highly expressed NOR1 could promote cisplatin resistance in HCC. After the nanoparticles entered the endosome, its acidic environment helped the degradation of core components and promoted the release of cisplatin and NOR1 shRNA, which synergistically promoted the cisplatin resistance of HCC cells into cisplatin sensitivity (Huang et al., 2023). Common chemotherapeutic drug nanoparticles have been listed in the reference (Jia et al., 2022).

### 4.2 Molecularly targeted therapy

As we all know, sorafenib and lenvatinib were the first-line targeted drug for advanced HCC, but sorafenib and lenvatinib resistance often occurs within 6 months of clinical application, resulting in ineffective or reduced effectiveness of treatment (Llovet et al., 2018). To address this drug resistance problem, coupling galactose on the surface of nanoparticles improves the efficiency of targeted delivery of nanoparticles to the liver, and at the same time improves the anti-hepatoma effect of sorafenib loaded with nanoparticles (Tunki et al., 2019). Further, the researchers prepared polylactic acid-glycolic acid - D-α-tocopheryl polyethylene glycol 1000 succinate nanoparticles (SPTNs) loading sorafenib. *In vivo and in vitro* experiments showed that the uptake of SPTNs by HCC cells increased by 1.6 times, and the particles could inhibit the expression of multidrug resistance-related genes in HCC cells, which showed a good antitumor effect (Tang et al., 2022). The exosome system including multiple siRNAs, SP94 peptide, U1-A’s n-terminal RNA recognition motif and exosomal membrane protein Lamp2b has been confirmed to enhance sorafenib-induced ferroptosis and inhibit sorafenib resistance (Li et al., 2022). Researchers have prepared Pullulan self-assembled nanoparticles that target HCC cells. The particles are coupled with stearic acid to increase the hydrophobicity of the particles, are biodegradable, and could effectively capture 95.6% sorafenib (Chirayil and Kumar, 2022). From the perspective of molecular mechanism, sorafenib resistance to HCC involves many regulations such as epigenetics, regulated cell death, and TME (Tang et al., 2020). Therefore, specifically targeting single or multiple regulation processes could help improve resistance to sorafenib. For example, liposomes carrying paclitaxel and sorafenib not only improve the anti-HCC efficacy of the single drug but also reduce multidrug resistance (Lei et al., 2019). Sorafenib and doxorubicin were encapsulated in the shell and nucleus of nanoparticles targeted by transferrin ligands, respectively, to synergically kill HCC cells through DNA insertion and inhibit the proliferation signaling pathways in HCC cells (Malarvizhi et al., 2014).

### 4.3 Immunotherapy

Tumor immunotherapy includes the use of cancer vaccines, cell therapy, immune checkpoint inhibitors, monoclonal antibodies, and small molecule inhibitors (Llovet et al., 2022). However, some patients with tumor immunotherapy show no response after achieving significant results in a short period, which is attributed to immune system resistance (Salani et al., 2022). For example, the surface of tumor cells expresses the programmed cell death protein 1 ligand (PD-L1), which can cause the function of cytotoxic T cells to be inhibited. Abnormally altered genes in tumor cells can reduce drug uptake or promote drug efflux, or the conformation of tumor targets can be changed to cause drug resistance (Heinrich et al., 2020). With the understanding of the mechanism of drug resistance to tumor immunotherapy, the following nanotechnology-based strategies have been proposed to improve the efficacy of tumor immunotherapy, such as generating neoantigens, tumor-specific antigens, immunomodulatory mRNA vaccines, mRNA-encoded antibodies and vaccines, and protein replacement derived from defective gene code products, etc (Sun et al., 2023). The mechanisms of these strategies and their applications in the treatment of HCC have been described in the literature and will not be detailed here.

The TME of HCC consists of tumor cells, extracellular matrix, and stromal cells, the latter of which include fibroblasts, endothelial cells, and immune cells. The TME is an important factor affecting HCC development such as immunity and drug resistance (Wu J. et al., 2023). The inhibitory immune microenvironment, tumor angiogenesis induced by local hypoxia, and tumor antigen loss or mutation promote the immune escape of HCC (Laface et al., 2023). Therefore, some nanodrug delivery systems that respond to external stimuli such as ultrasonic stimulation, heat, pH, and light have shown great potential in regulating TME (Li et al., 2020). The regulatory strategies of the nanodrug delivery system for HCC TME mainly include transforming the cell state and targeting TME-related specific markers or signaling pathways. GNPs-Dox-Lac, an ECM-sensitive nano-delivery system consisting of gelatin nanoparticles (GNPs) and the prodrug doxorubicin lactose (DOX-LAC), could specifically target the salivary glycoprotein-1 receptor (asgpr1) of hepatocytes. GNPs are degraded by MMP2 to facilitate the penetration of Dox into tumors. The pH response characteristics are conducive to the release of free Dox in HCC. The sensitivity of ECM makes the tumor inhibition rate of this nanoparticle reach more than 90% *in vivo* (Liu et al., 2018). Nanoparticles that modulate the immune system and TME of HCC have been listed in the reference (Huang et al., 2022). However, the regulatory effects of nanodrug delivery systems on myeloid-derived suppressor cells and tumor-associated neutrophils in HCC have been poorly studied. Multitarget nanodelivery systems that could simultaneously target multiple TME components in HCC are promising.

### 4.4 Interventional nanotherapy

Interventional therapy is an intra-vascular and natural cavity intervention, such as the common transarterial chemoembolization (TACE) and radiofrequency ablation (RFA). Using the reactivity of nanoparticles to external stimuli such as ultrasound and heat, the combination of interventional therapy and these properties forms interventional nanotherapeutics. The nano-drug delivery system could improve the targeting and stability of chemotherapy drugs in TACE and improve the efficacy of anti-HCC (Wu S. et al., 2023). TACE combined with a sorafenib nanodrug delivery system can effectively reduce the toxicity of monotherapy and improve the disease control rate (Su, 2021). The thermal effect of RFA combined with the thermal response characteristics of nanomaterials could influence the release of chemotherapy drugs. The combination of ThermoDox encapsulated by lysophospholipids and RFA is beneficial to the release of doxorubicin. The phase transition temperature of the liposomes is about 40°C. When the liposomes are heated, doxorubicin is released by lipid pores or enhanced lipid permeation. However, RFA provides a local temperature environment of 42°C, which can effectively heat the liposomes (Rahim et al., 2021). While the short-release temperature window led to the failure of clinical trials. Given the great potential of interventional nanotherapy in the treatment of HCC, researchers are continuing to improve and explore. At present, clinical studies on the treatment of HCC with interventional nanotherapies are going (Chavda et al., 2023).

### 4.5 Other therapies

The gas levels of the tumor microenvironment are different from those of normal tissue. Therefore, the specific characteristics are an ideal endogenous treatment strategy. For instance, NanoNO can sustainably release and effectively deliver NO gas to HCC cells to normalize HCC vasculature (Sung et al., 2019). Gas therapy-related nanomaterials can not only overcome drug resistance in tumor therapy but also are not affected by tissue depth, which is an effective strategy to achieve accurate cancer therapy.

Radiotherapy is a palliative therapy to improve the quality of life of patients with advanced HCC, and it is mainly used in combination with other therapies. In the nanotechnology of HCC treatment, the development of nano-radiosensitizer is the main. Xiao et al. found that targeted nano self-assembled Pt STNA containing platinum drugs can enhance ROS levels in HCC cells under X-ray irradiation and thus synergistically play the role of radiosensitizer and chemotherapy (Xiao et al., 2022).

Photodynamic/sonodynamic/photothermal therapy (PDT/SDT/PTT) is a new therapy in the treatment of HCC for its advantages of being non-invasive and accurate. Photodynamic therapy mainly uses a specific wavelength of laser irradiation to stimulate the photosensitizer absorbed by the tissue, resulting in cytotoxic effects. Sonodynamic therapy uses ultrasound to penetrate biological tissues, especially focused ultrasound can focus sound energy on deep tissues without trauma, and activate some sound-sensitive drugs (such as hematoporphyrin) to produce anti-tumor effects. PTT is a new tumor treatment method, which is to inject nanoparticles into tumor tissue, and then use laser irradiation of nanoparticles to produce photothermal effect, thereby killing tumor cells. Copper sulfide nanoparticles coated with hybrid film have a photothermal conversion effect under near-infrared irradiation, which can be used for photothermal therapy of HCC (Ji et al., 2020). At present, it is more common to combine other treatment methods with PDT/SDT/PTT to improve anti-tumor efficacy due to their limited value for metastatic tumors and local recurrence (Li K. et al., 2021; Fan et al., 2021). Common combination drugs are chemotherapy drugs such as doxorubicin, sorafenib, and gene therapy. For example, Au@MSN - PGEA@SF@P53 nanoparticles added with the p53 gene not only showed the synergistic anti-HCC efficacy of PTT and sorafenib but also showed the high transfection efficiency and therapeutic effect of the p53 gene (Chen et al., 2018). The CAR-T cell membrane, which specifically recognizes GPC3+ HCC cells, was coated on mesoporous silica containing IR780 nanoparticles. In this way, the nanomaterial prepared has a strong targeting ability and photothermal anti-HCC effect (Ma et al., 2020). Nanobubbles (RSL3@O2-ICG NBs) attached to the ultrasonic sensitizer indocyanine Green (ICG) and loaded with RSL3 have been shown to enhance the sonodynamic therapeutic effect and promote ferroptosis in HCC cells (Chen et al., 2022).

### 4.6 Combination therapy

Combining the advantages of nanodelivery systems allows for the combination of multiple HCC therapies to maximize efficacy and reduce the toxic side effects of monotherapies. Radiosensitization and PDT/SDT/PTT mentioned above are often combined with other treatments. The iridium-based photosensitizer is combined with sorafenib (IPS) in the form of self-assembly. Under the action of 532 nm laser, the uptake of this particle by HCC cells is increased, and the intracellular ROS is increased to enhance the anti-HCC effect (Abuduwaili et al., 2022). In addition, combining multiple therapeutic strategies of the same therapy with nanodrug delivery systems, such as BisCCL2/5i, a biospecific antibody encoding chemokine ligand 2 (CCL2) and CCL5, could not only inhibit the accumulation of tumor-associated macrophages and reverse immunosuppression but also increases the sensitivity of HCC to PD-1 ligand inhibitor (PD-Li) (Wang et al., 2021). Combination therapy has shown great potential in animal models of HCC, and efforts are underway to advance this therapy into preclinical trials.

## 5 Conclusion and perspectives

Here, we reviewed strategies for improving the efficacy of HCC drugs with nano-delivery systems and their applications in HCC treatment. Nanoparticles can protect drugs from degradation and improve their targeting to improve the efficiency of HCC treatment. At present, nanodrug delivery systems show great potential in the treatment of HCC, especially immunotherapy, because they can effectively enhance the immune response. However, the regulatory effect of nanoparticles on tumor immune cells, such as tumor-associated neutrophils, has not been fully clarified, and these cells are key to improving HCC immunotherapy, requiring further efforts by researchers.

In addition, there are still some problems that need to be solved in the treatment of HCC. First, there is a need for better research on whether the nanoparticles and their formulations cause unnecessary immune responses and toxic side effects *in vivo*, such as off-target effects of mRNA vaccines loaded with lipid nanoparticles, even when an intramuscular injection is performed (Hassett et al., 2019). Therefore, we should also focus on improving the effectiveness of nanodrug delivery systems and exploring the best delivery routes. Secondly, to further explore and design a multi-target nanodrug delivery system that can simultaneously target multiple therapeutic targets in HCC, it has a better therapeutic effect. Finally, the clinical conversion efficiency of nano drug delivery systems is still low, which needs to be solved. At present, there are many pieces of research on nano drug delivery systems in HCC, but few have entered the clinical trial stage, and even fewer have been approved for clinical application, the product with two siRNAs against the kinesin spindle protein (KSP) and vascular endothelial growth factor (VEGF) entered in clinical trial (NCT 00882180) (Tabernero et al., 2013). In conclusion, nano-delivery systems have broad prospects in the treatment of HCC, and the efficacy and prognosis of HCC are expected to make significant progress.
